# Case Report: Third-degree atrioventricular block and respiratory failure caused by clozapine poisoning

**DOI:** 10.3389/fphar.2025.1572578

**Published:** 2025-07-18

**Authors:** Qiaoxin Tian, Ruikai Shang, Yuru Liu, Hongyu Liu, Yingying Zheng, Xiangdong Jian

**Affiliations:** ^1^ Department of Occupational and Environmental Health, School of Public Health, Cheeloo College of Medicine, Shandong University, Jinan, Shandong, China; ^2^ Department of Poisoning and Occupational Diseases, Emergency Medicine, Qilu Hospital of Shandong University, Cheeloo College of Medicine, Shandong University, Jinan, Shandong, China; ^3^ School of Nursing and Rehabilitation, Cheeloo College of Medicine, Shandong University, Jinan, Shandong, China

**Keywords:** clozapine, poisoning, atrioventricular block, rhabdomyolysis, respiratory failure

## Abstract

**Background:**

This case report details the management of a patient who presented with third-degree atrioventricular block and rhabdomyolysis secondary to clozapine intoxication.

**Case summary:**

The patient was a 55-year-old man who took 100 tablets of clozapine and was transferred to our hospital from a lower-level hospital for treatment. Upon arrival at our hospital, he was in a coma and was assisted with mechanical ventilation. Upon admission, based on the results of toxicological tests and laboratory examination; computed tomography, magnetic resonance imaging, and echocardiography findings, and the patient’s clinical manifestations, the diagnosis of third-degree atrioventricular block and rhabdomyolysis due to excessive intake of clozapine was confirmed. The patient received comprehensive treatment, including blood purification, organ protection, nutritional support, and cardiac rate enhancement. The patient was clinically cured and discharged. Clozapine-induced central nervous system inhibition can be dose-dependent, thus leading to coma and organ damage at high doses. Considering that no specific antidotes are available, cases involving clozapine toxicity require careful management. In this instance, beyond the central nervous system and respiratory depression, the patient also exhibited third-degree atrioventricular block and rhabdomyolysis, which warrant significant attention.

**Conclusion:**

Many patients with clozapine poisoning have been admitted to our department. Clozapine poisoning mostly causes symptoms such as accelerated heart rate, but in our patient’s case, third-degree atrioventricular block and rhabdomyolysis symptoms occurred unusually. For clozapine poisoning, timely and appropriate management is crucial for the recovery of patients.

## 1 Introduction

Clozapine, with the molecular formula C_18_H_19_ClN_4_, was the first atypical antipsychotic drug, initially synthesized in 1959 (Khokhar et al., 2018). It exerts complex pharmacological effects by potently blocking 5-hydroxytryptamine receptors and dopamine receptors in the brain (Khokhar et al., 2018). Additionally, it possesses anticholinergic, antihistaminergic, and anti-α-adrenergic properties and modulates glutamatergic and γ-aminobutyric acid systems (Olney et al., 1999; Wenthur and Lindsley, 2013). Despite being withdrawn from the market in 1970 due to cases of agranulocytosis, clozapine was reapproved by the United States Food and Drug Administration in 1990 (Hippius, 1999). To this day, the precise mechanism by which clozapine is highly effective in treating refractory schizophrenia has not been fully clarified. Clozapine has a weak affinity for D2 receptors, but it can bind more closely to 5-hydroxytryptaminergic, α -adrenergic, metabolic glutaminergic, and muscarinic receptors. In addition, it has neuroprotective, anti-proliferative, and anti-inflammatory actions. The metabolite of clozapine, *N*-desmethylclozapine, can act as a positive allosteric modulator of the muscarinic M1 receptor and an agonist of the M4 receptor. This mechanism of action is likely to contribute to the unique therapeutic effect of clozapine. Although its use is associated with adverse reactions affecting the respiratory, digestive, and circulatory systems, clozapine remains widely prescribed because clozapine has significant advantages over other dopamine receptor blocker antipsychotic drugs in managing refractory schizophrenia: it is more effective in reducing positive symptoms; reducing the risk of recurrence/hospitalization, suicidal behavior, substance abuse, and aggressive behavior; enhancing social functions including employment; and reducing the risk of death (Correll, 2025). Therefore, its clinical use has been re-approved in China and abroad, with a utilization rate of 39.0% in China (Jingping and Shenxun, 2015). Cases of acute clozapine poisoning, often due to overdose or accidental ingestion, occur frequently. This report details a case of acute clozapine poisoning confirmed through toxicological testing, where the patient developed third-degree atrioventricular block and respiratory failure.

## 2 Case description

Ethical approval was obtained from the Ethics Committee of Qilu Hospital of Shandong University, and informed consent was obtained from the patient.

A 55-year-old man with schizophrenia, who had been taking oral clozapine, was transferred from a low-level hospital to our hospital with a ventilator on 13 February 2024, for “altered consciousness for 2 days” On admission, physical examination showed a temperature of 36.9°C, heart rate of 49 beats/min, respiratory rate of 15 breaths/min, blood pressure of 132/61 mmHg, and SpO_2_ of 99%. He was in a sedated and analgesic state (Glasgow Coma Scale, 1-1–3, 5) and was on mechanical ventilation. His pupils were approximately 2.0 mm in diameter, with sluggish light reflexes. Lung examination revealed coarse breath sounds without wet rales. No pathological murmur was detected in the heart. The abdomen was soft, and the liver and spleen were not palpable. No spinal or limb deformities were noted, and physiological reflexes were present without pathological signs.

## 3 Diagnostic assessment

**FIGURE 1:**
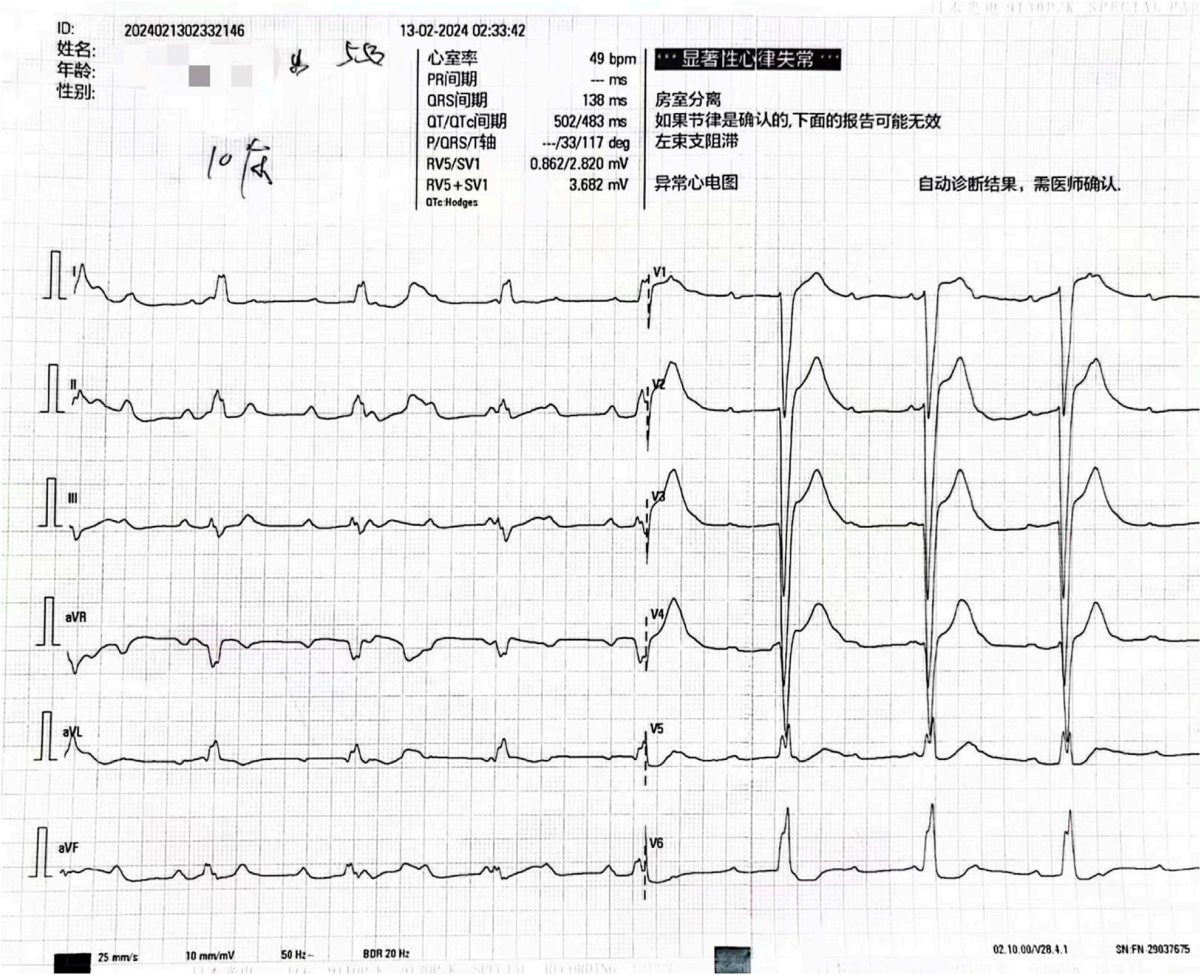
Electrocardiogram showed third-degree atrioventricular block.

Initial diagnoses included altered consciousness, respiratory failure, and suspected drug poisoning. After admission, various examinations were performed promptly. Toxicology test results revealed a clozapine concentration of 1800.00 ng/mL. An electrocardiogram indicated third-degree atrioventricular block (Figure 1), and laboratory test results indicated creatine kinase levels of 9255 U/L, creatine kinase-myocardial band (CK-MB) levels of 29.20 ng/mL, myoglobin levels of 368.00 ng/mL, and cardiac troponin levels of 237.72 ng/L. The patient was diagnosed with acute clozapine poisoning, respiratory failure, third-degree atrioventricular block, and rhabdomyolysis.

## 4 Intervention

**FIGURE 2:**
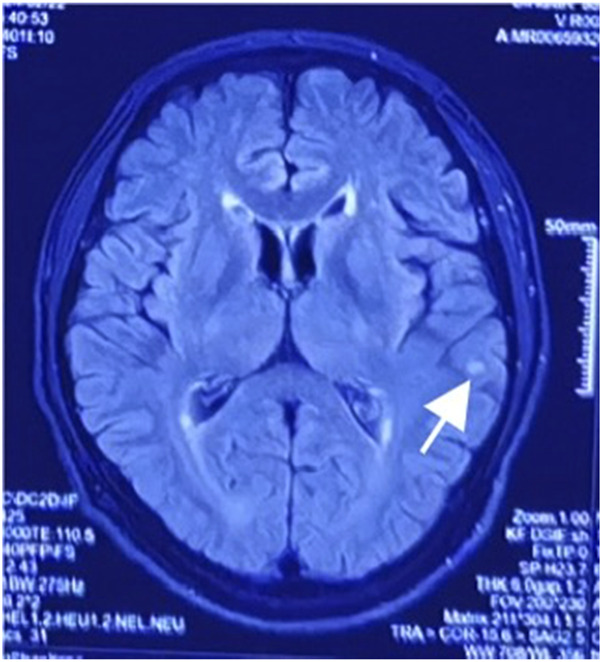
Magnetic resonance imaging examination revealed a few ischemic lesions in the brain.

**FIGURE 3:**
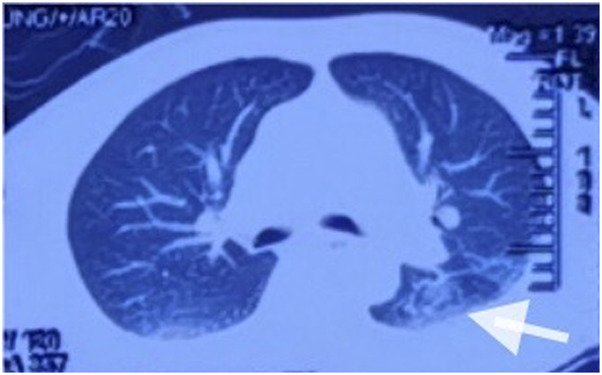
Chest computed tomography scan revealed slight pneumonia in both lungs.

**FIGURE 4:**
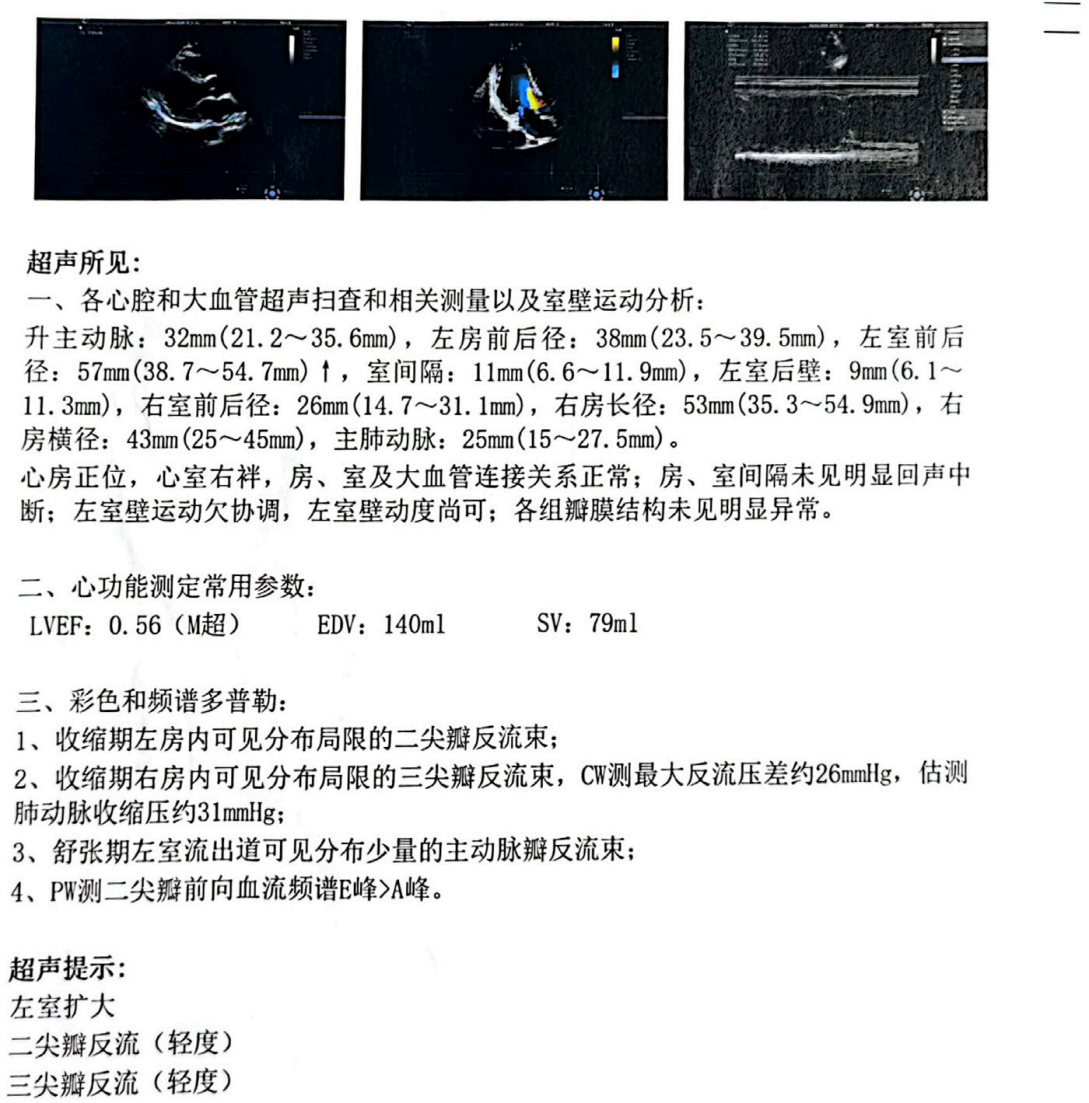
Echocardiographic report.

After admission, continuous ventilator-assisted ventilation was provided. Considering that the patient’s inflammatory indicators such as PCT (0.159 ng/mL), NEU% (78.6%), and LYM% (13.20%) were abnormal; presence of apoptosis and inflammation in both lungs; and excessive concentration of clozapine, hemoperfusion and continuous renal replacement therapy were required. Therefore, we used flucloxacillin for anti-infection. Meanwhile, we used diuretics to promote excretion and intravenous injection of fat emulsion for detoxification and nutritional support. We invited cardiology experts for consultation who recommended the use of a temporary pacemaker. The patient was in a coma. After repeated communication, the patient’s family insisted on conservative treatment and signed a document refusing the installation of a pacemaker (Liang et al., 2011). By the third day of admission, his heart rate had decreased to 32 beats/min. A temporary cardiac pacemaker was recommended, but the patient’s family declined. On the sixth day, sedation was reduced, and he was weaned off the ventilator. On the seventh day, the tracheal tube was removed, and furosemide and flucloxacillin were discontinued. The creatine kinase level decreased to 122 U/L, and the CK-MB decreased to 4.5 ng/mL. After the patient regained consciousness, we inquired about the medical history. Before the patient fell into a coma, 100 tablets of clozapine were taken orally, and no other drugs were used simultaneously. This information was consistent with the results of the toxicological tests. On the eighth day, his spontaneous heart rate increased, allowing a gradual reduction in isoproterenol. By the ninth day, head magnetic resonance imaging showed a few ischemic and degenerative foci in the brain (Figure 2), and chest computed tomography images showed a few areas of pneumonia in both lungs (Figure 3). An echocardiogram indicated left ventricular dilation, with a posterior diameter of 57 mm (normal value: 38.7–54.7 mm) (Figure 4). On the 12th day, after discontinuing isoproterenol, his spontaneous heart rate stabilized at 55 beats/min, with no symptoms such as blackouts, dizziness, or syncope. The patient was discharged on the 14th day.

## 5 Follow-up

Follow-up phone calls confirmed that he remained self-sufficient in daily life and had no significant abnormal manifestations.

## 6 Discussion

Clozapine is recommended for use at a blood concentration of 300–420 ng/mL. When its blood concentration exceeds 600 ng/mL, patients may experience adverse reactions (Rajkumar et al., 2013), while toxic symptoms typically develop at concentrations above 1,000 ng/mL (Haack et al., 2003). Clozapine poisoning primarily manifests as central nervous system depression, respiratory depression, and cardiovascular dysfunction (Krämer et al., 2010). At high doses, the dose-dependent central nervous system inhibition often leads to coma. Severe clozapine poisoning may cause respiratory depression, characterized by changes in breathing rhythm, cyanosis of the lips, and apnea. Aspiration pneumonia caused by toxic coma is a leading cause of death in clozapine-related cases (Krämer et al., 2010). Therefore, clinicians must closely monitor comatose patients for respiratory complications. Acute clozapine poisoning commonly affects the cardiovascular system, manifesting as hypertension, tachycardia, and prolonged QTc interval (Krämer et al., 2010). The symptoms of acute clozapine poisoning include dysarthria, myoclonus, bradykinesia, tremors, nausea, vomiting, dry mouth, excessive salivation, and fever (Krämer et al., 2010). Studies have shown that psychotropic drugs may cause an increase in troponin, which was also observed in this case (Wan et al., 2025). The diagnosis relies primarily on medical history, physical examination, and blood concentration testing. A low blood concentration of clozapine does not rule out acute clozapine poisoning, as there have been reports of poisoning occurring within the therapeutic window. In this case, the patient presented with third-degree atrioventricular block and rhabdomyolysis in addition to central nervous system and respiratory depression. Complete atrioventricular block induced by clozapine is rare, with only two cases reported in the literature (Gabeler and van Miltenburg, 2011; Türe et al., 2019). This condition may result from clozapine-mediated inhibition of sinus node depolarization and reduced adrenergic sensitivity (Krobert et al., 2006). Clozapine is cardiotoxic and has been linked to myocarditis and cardiomyopathy (Siskind et al., 2020). Clozapine-induced reduction of selenium, an important antioxidant involved in myocardial recovery, impairs the repair of the damaged conduction system and causes a conduction block (Vaddadi et al., 2003). In this case, myocardial damage was evident from an elevated serum high-sensitivity troponin level of 237.72 ng/L at admission (normal value <17.5 ng/L) and left ventricular dilation observed on echocardiography. When third-degree atrioventricular block occurs, it is essential to promptly identify the cause, manage infections, and correct electrolyte disorders. Cardiac pacing therapy should be administered when hemodynamic instability is caused by a slow ventricular rate. However, the patient’s family refused temporary cardiac pacing in this case.

Rhabdomyolysis occurs when damage to muscle cells results in the release of cellular contents into the bloodstream. Rhabdomyolysis is usually defined as creatine kinase >1000 IU/L or CK > 5 times the normal upper limit (Stahl et al., 2020). Early intervention is critical for preventing life-threatening complications, such as acute kidney injury. In this case, the peak creatine kinase level was 9255 IU/L; therefore, the diagnosis of rhabdomyolysis was confirmed. The mechanism by which clozapine causes rhabdomyolysis is also unclear, but all muscle injuries follow a common pathway: the muscle cell membrane is directly destroyed or muscle cell energy is depleted (Bosch et al., 2009), and free calcium enters the cell and activates the protease and apoptosis pathways (Giannoglou et al., 2007). The production of reactive oxygen species leads to mitochondrial dysfunction and, ultimately, cell death (Giannoglou et al., 2007). Previously, we reported a case of rhabdomyolysis caused by olanzapine poisoning, which may have been related to the muscular toxicity of the drug and long-term muscle compression caused by coma (Yu et al., 2021).

Currently, no specific antidote for clozapine poisoning exists. Symptomatic supportive treatment remains the mainstay, and maintaining the stability of respiration and circulation is important (Levine and Ruha, 2012). When patients consume large amounts of oral medicine and have serious complications, toxic symptoms can be alleviated by blood purification (Kirilochev et al., 2024). In cases of unexplained bradycardia encountered in clinical practice, the possibility of cardiac dysfunction due to high-dose or long-term oral administration should be considered.

## Data Availability

The original contributions presented in the study are included in the article/supplementary material, further inquiries can be directed to the corresponding author.
